# Population Diversity Matters: Heterogeneity of Biopsychosocial Pathways from Socioeconomic Status to Tobacco Use via Cerebral Cortical Volume in the ABCD Study

**DOI:** 10.31586/jcn.2025.1132

**Published:** 2025-01-23

**Authors:** Shervin Assari, Hossein Zare

**Affiliations:** 1 Department of Internal Medicine, Charles R. Drew University of Medicine and Science, Los Angeles, CA, United States; 2 Department of Family Medicine, Charles R. Drew University of Medicine and Science, Los Angeles, CA, United States; 3 Department of Urban Public Health, Charles R. Drew University of Medicine and Science, Los Angeles, CA, United States; 4 Marginalization-Related Diminished Returns (MDRs) Center, Los Angeles, CA, United States; 5 Department of Health Policy and Management, Johns Hopkins Bloomberg School of Public Health, Baltimore, MD, United States; 6 School of Business, University of Maryland Global Campus (UMGC), Adelphi, MD, United States

**Keywords:** Racial Disparities, Socioeconomic Status, Cortical Volume, Tobacco Use, Adolescent Brain Development, Marginalization-Related Diminished Returns, ABCD Study

## Abstract

**Background:**

Most neuroscience research has predominantly focused on White, middle-class populations, leading to gaps in understanding how socioeconomic status (SES) influences brain development and health behaviors in racially diverse groups. Tobacco use, a major public health concern, is influenced by both family and neighborhood SES, with early initiation during adolescence predicting long-term health outcomes. The Adolescent Brain Cognitive Development (ABCD) study provides a unique opportunity to examine racial disparities in the pathways from SES to brain development and behavior, especially through the lens of Marginalization-Related Diminished Returns (MDRs), where the effects of SES are attenuated for minority groups.

**Objective:**

This study investigates racial variation in the associations between SES, cerebral cortical volume, and tobacco use initiation, comparing Black and White youth over 4-6 years of follow-up.

**Methods:**

Data from the ABCD study were analyzed to assess pathways from family income to adolescents’ cortical volume via the needs-to-income ratio, and from cortical volume to tobacco use initiation. Structural equation modeling was used to evaluate these pathways, stratified by race, with a focus on comparing Black and White participants. Covariates included family and neighborhood SES, demographic factors, and baseline behavioral measures.

**Results:**

We found that the positive association between income (via the needs-to-income ratio) and total cortical volume was significantly weaker for Black youth compared to White youth. Additionally, the link between larger total cortical volume and reduced risk of tobacco initiation was also weaker in Black adolescents. These findings were consistent over 4-6 years of follow-up, suggesting that Black youth experience diminished returns from higher SES in terms of brain development and behavioral outcomes.

**Conclusions:**

Our findings highlight significant racial disparities in the pathways from SES to brain development and tobacco use initiation, supporting the Marginalization-Related Diminished Returns (MDRs) framework. While higher SES is associated with larger cortical volumes and lower tobacco use risk in White youth, these associations are attenuated in Black adolescents.

## 1. Introduction

Socioeconomic status (SES) significantly impacts brain development, which in turn influences behaviors such as tobacco use [1-6]. Higher SES [7-10] is typically associated with better access to resources, nutrition, educational opportunities, and lower stress, [11-20, all of which contribute to increased cortical volume [21-28]. In addition to its correlation with SES, larger cortical volume is linked to enhanced cognitive functions such as emotion regulation, decision-making, planning, and inhibitory control, which are critical for avoiding risk behaviors like tobacco use during adolescence.

However, most neuroscience research has predominantly focused on individuals racialized as White, primarily from middle-class populations. This narrow focus leaves substantial gaps in understanding how the pathways from social environments to brain development and behavior vary across racially diverse and historically marginalized groups who have experienced different social and economic conditions. The lack of diversity in research limits the generalizability of findings, particularly regarding how SES interacts with brain development and health behaviors in underrepresented populations. Addressing these gaps is crucial for informing interventions and policies aimed at reducing health disparities.

The Adolescent Brain Cognitive Development (ABCD) study [29-34] offers an unprecedented opportunity to explore these variations, as it includes a diverse dataset with comprehensive social, behavioral, and neurobiological measures. This rich data enables a thorough analysis of the pathways from SES to brain structure—specifically total cortical volume—and how these pathways relate to behaviors such as tobacco use initiation across different racial and ethnic groups.

An increasing body of research suggests that the associations between SES, brain structure, and behavior differ across racial and ethnic lines [35-40]. A key aspect of brain development influenced by SES is cerebral cortical volume, which is associated with cognitive functions crucial for self-regulation. Higher SES is linked to larger cortical volumes, which in turn support better emotion regulation, decision-making, and inhibitory control—skills essential for reducing the likelihood of engaging in risky behaviors like tobacco use. Adolescence is a particularly critical period for brain development, as early tobacco use during this stage often predicts long-term negative health outcomes.

For minoritized and racialized youth, particularly Black adolescents, the relationship between SES and health-related outcomes may be attenuated, a phenomenon described as Marginalization-Related Diminished Returns (MDRs) [41-52]. This framework posits that due to systemic inequalities, chronic social stressors, and historical marginalization, the benefits of higher SES on brain development and health behaviors are often weaker for racialized and minoritized groups compared to their socially privileged White counterparts [38,53-58].

Tobacco use among adolescents is strongly shaped by social factors, particularly family and neighborhood SES [59-61]. Adolescents from lower SES backgrounds or disadvantaged neighborhoods are at higher risk of initiating tobacco use, underscoring the importance of understanding how these social determinants vary by race and ethnicity. Emerging evidence suggests that the strength of these pathways is not uniform across populations.

In this paper, we leverage data from the ABCD [29-34] study to examine how the pathways from family and neighborhood SES to tobacco use via cerebral cortical volume differ across groups defined by racialization. Specifically, we hypothesize that the positive associations between SES and cortical volume, as well as the protective associations between total cortical volume and tobacco initiation, will be weaker for adolescents racialized as Black compared to those racialized as White, who have historically retained social privilege. Importantly, this weakened association is not rooted in biological differences but in the sociology of race, reflecting historical, social, and contextual factors such as trauma and systemic inequality. This study aims to elucidate the complex interplay between social factors, brain development, and health behaviors, while addressing critical gaps in understanding population diversity and the impacts of racialization on these processes.

## 2. Methods

### 2.1. Settings and Design

This study utilized data from the Adolescent Brain Cognitive Development (ABCD) Study, a large-scale, longitudinal research initiative designed to examine brain development and child health in the United States. The ABCD study recruited over 11,000 children aged 9-10 years from 21 sites across the country, using a multi-stage probability sampling method to ensure a diverse and representative sample. Data collection involved a combination of neuroimaging, behavioral assessments, and questionnaires completed by both children and their parents. The current analysis leverages cross-sectional baseline data from the ABCD study, focusing on brain structure, socioeconomic factors, and demographic variables.

### 2.2. Sample and Sampling

The initial sample for the ABCD study included 11,878 children. For this analysis, we restricted the sample to children identified by their parents as either Black or White, consistent with our focus on racial disparities. To ensure reliable estimates of brain structure, children with missing or poor-quality neuroimaging data were excluded from the sample.

### 2.3. Eligibility for the Current Analysis

Eligibility for inclusion in the current analysis required that children meet the following criteria: (1) aged 9-10 years at baseline, (2) identified as Black or White, (3) completed parental questionnaires on socioeconomic factors, and (4) had complete neuroimaging data for total cortical volume. Children with missing key demographic or socioeconomic data (e.g. race) were excluded from the analysis.

### 2.4. Measures

#### Mediator (Total Cortical Volume):

The primary outcome of interest was total cortical volume, measured using MRI data collected as part of the ABCD study’s neuroimaging protocol. Cortical volume was calculated by summing the volumes of cortical gray matter across both hemispheres, using FreeSurfer software for brain image processing.

#### Predictor (Family Income to Needs Ratio):

The key independent variable was family **income to needs ratio**, measured at the level of family, considering household income and family size, and poverty ration. This was treated as a continuous variable, representing higher SES.

#### Outcome (Tobacco Use Initiation):

Tobacco use initiation was defined as the first reported use of any tobacco product, including cigarettes, e-cigarettes, and other tobacco-related products, at any follow-up assessment during the study period.

### 2.5. Covariates

We controlled for several demographic variables that could influence brain development, including the child’s age, sex, and parental marital status. Marital status was categorized as married or not married. Age and sex were included to account for normal developmental differences in cortical volume across children.

### 2.6. Statistical Analysis

We employed Structural Equation Modeling (SEM) to test the associations between family SES (income to needs ratio) and tobacco initiation, with total cortical volume as a potential mediator and race as a moderator. Our analyses were conducted using SEM in Stata, and we used maximum likelihood estimation to account for missing data. Model fit was assessed using standard indices such as the Comparative Fit Index (CFI) and Root Mean Square Error of Approximation (RMSEA). Significance was evaluated at p < .05.

### 2.7. Ethics

The ABCD study received approval from the Institutional Review Boards (IRBs) at each of the 21 data collection sites. Informed consent was obtained from all parents or legal guardians, and assent was obtained from children before participation. This study’s secondary analysis of de-identified ABCD data was exempted from a full IRB review by the Charles R. Drew University of Medicine and Science.

## 3. Results

### 3.1. Descriptive Data

Table 1 provides the descriptive statistics for the key study variables. The average age of the children in the sample was 9.48 years (SE = 0.005), with a 95% confidence interval ranging from 9.47 to 9.49. In terms of race, 72.9% of the sample identified as White (SE = 0.006), and 27.1% identified as Black (SE = 0.006). The sample was fairly balanced by gender, with 47.6% of participants identifying as female (SE = 0.007) and 52.4% as male (SE = 0.007). Regarding the marital status of the household, 34.0% of children lived in an unwed household (SE = 0.006), while 66.0% lived in a married household (SE = 0.006).

### 3.2. Summary of Multigroup Structural Equation Model

**Figure 1.**
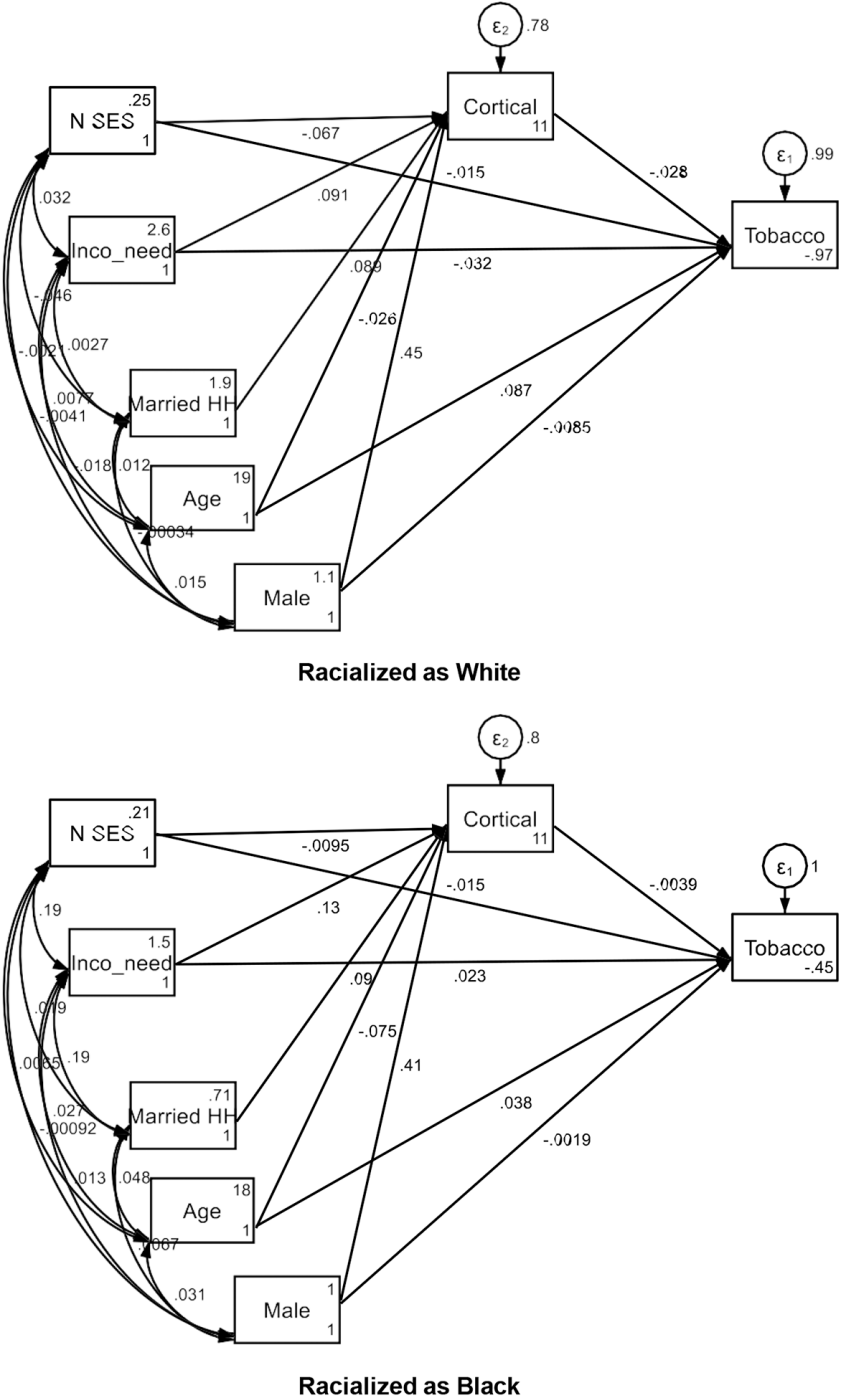
Summary of structural equation models Note: Data Source: ABCD Study; HH: Household; N SES: Neighborhood Socioeconomic Status; Tobacco: Tobacco Initiation; Cortical: Total Cortical Volume

Table 2 and Figure 1 present the results of the multigroup structural equation modeling for the pathways from socioeconomic status (SES) to tobacco use initiation via total cortical volume, stratified by racialization as either White or Black. Overall, the results highlight the differences in the pathways from SES to tobacco use initiation via total cortical volume between adolescents racialized as Black and White.

For adolescents racialized as White, the analysis indicates several significant predictors of tobacco use initiation. Total cortical volume is negatively associated with tobacco use initiation (B = −0.028, SE = 0.012, 95% CI = [−0.053, −0.004], p = 0.022), suggesting that higher cortical volume is linked to lower tobacco use risk. Age is positively associated with tobacco use initiation (B = 0.087, SE = 0.011, 95% CI = [0.065, 0.108], p < 0.001), indicating that older adolescents are more likely to initiate tobacco use. The income-to-need ratio also negatively predicts tobacco use initiation (B = −0.032, SE = 0.012, 95% CI = [−0.054, −0.009], p = 0.006), suggesting that better socioeconomic conditions reduce the risk of tobacco initiation. However, low neighborhood income does not significantly predict tobacco use initiation (B = −0.015, SE = 0.011, 95% CI = [−0.037, 0.008], p = 0.197).

Regarding total cortical volume for adolescents racialized as White, age is negatively associated (B = −0.026, SE = 0.010, 95% CI = [−0.045, −0.007], p = 0.008), and male gender positively predicts larger cortical volume (B = 0.450, SE = 0.009, 95% CI = [0.433, 0.467], p < 0.001). The income-to-need ratio is positively associated with cortical volume (B = 0.091, SE = 0.010, 95% CI = [0.071, 0.110], p < 0.001), while low neighborhood income is negatively associated (B = −0.067, SE = 0.010, 95% CI = [−0.087, −0.048], p < 0.001). The model intercept is significant (B = 10.902, SE = 0.206, 95% CI = [10.497, 11.306], p < 0.001).

For adolescents racialized as Black, the findings indicate that total cortical volume does not significantly predict tobacco use initiation (B = −0.004, SE = 0.023, 95% CI = [−0.048, 0.040], p = 0.865). Age shows a trend toward significance (B = 0.038, SE = 0.020, 95% CI = [−0.002, 0.077], p = 0.063), while the male gender and income-to-need ratio do not significantly predict tobacco use initiation (B = −0.002, SE = 0.022, 95% CI = [−0.045, 0.042], p = 0.931; B = 0.023, SE = 0.022, 95% CI = [−0.020, 0.067], p = 0.290). Low neighborhood income also does not significantly predict tobacco use initiation (B = −0.015, SE = 0.021, 95% CI = [−0.056, 0.027], p = 0.482). The intercept is not significant (B = −0.447, SE = 0.452, 95% CI = [−1.333, 0.439], p = 0.323).

For total cortical volume among adolescents racialized as Black, age negatively predicts cortical volume (B = −0.075, SE = 0.018, 95% CI = [−0.111, −0.040], p < 0.001). Male gender is positively associated with cortical volume (B = 0.413, SE = 0.017, 95% CI = [0.380, 0.445], p < 0.001). The income-to-need ratio positively predicts cortical volume (B = 0.129, SE = 0.020, 95% CI = [0.089, 0.168], p < 0.001), while low neighborhood income does not show a significant association (B = −0.009, SE = 0.019, 95% CI = [−0.048, 0.029], p = 0.626). The intercept is significant (B = 11.308, SE = 0.364, 95% CI = [10.594, 12.022], p < 0.001).

## 4. Discussion

Diversity in neuroscience research is essential for understanding how brain development and behavior may vary across different racial and ethnic groups. Stigmatizing this diversity not only perpetuates harmful stereotypes but also leads to inaccurate scientific conclusions and misguided policy decisions. The Adolescent Brain Cognitive Development (ABCD) study offers a unique opportunity to explore the complex interplay between social, environmental, and biological factors in a diverse sample, helping to address gaps in the literature. Failing to recognize this diversity could lead to the incorrect assumption that achieving socioeconomic (SES) equity alone would eliminate racial disparities in development and behavior. This belief overlooks the deeply rooted structural inequalities that affect minoritized and racialized populations and may result in the continuation of policies that do not address the broader social context contributing to these disparities.

The aim of this study was to examine racial variation in the pathways from family and neighborhood SES to tobacco use via cerebral cortical volume. We hypothesized that the positive associations between SES and total cortical volume, as well as between cortical volume and tobacco initiation, would be weaker for Black adolescents compared to White adolescents. This hypothesis was grounded in the framework of Marginalization-Related Diminished Returns (MDRs), which posits that minoritized and racialized groups experience fewer benefits from higher SES due to structural inequalities.

Our findings confirmed that the pathways from SES to brain structure and from brain structure to tobacco use were both weaker in Black adolescents compared to White adolescents. Specifically, we found that the link between income (through the needs-to-income ratio) and total cortical volume was significantly attenuated in Black youth. Similarly, the association between larger cortical volume and a lower likelihood of initiating tobacco use was also weaker in Black youth. These results were consistent over the 4-6 years of follow-up in the ABCD dataset.

These findings align with the existing Marginalization-Related Diminished Returns (MDRs) literature, which has demonstrated that the health and developmental benefits of higher SES are often diminished for racial and ethnic minorities. Our study adds to this body of work by showing that the weakened associations between SES, brain development, and behavior extend to neural structures like the cerebral cortex and health behaviors like tobacco use in adolescents.

Importantly, these diminished returns are not due to any biological inferiority or superiority of one racial group over another. Instead, they reflect the long-standing effects of structural racism, including segregation, social stratification, and systemic inequality. These structural inequities are deeply rooted in the historical legacies of slavery, Jim Crow laws, and ongoing forms of racial discrimination. These social conditions have created environments where even high SES does not afford the same protective benefits to Black individuals as it does to White individuals, due to the cumulative effects of stress, discrimination, and reduced access to resources.

Structural racism deeply impacts all aspects of life for individuals racialized as Black, and SES alone is not sufficient to protect against its pervasive effects [62-67]. While higher SES provides certain advantages, such as access to better resources and opportunities, it does not shield Black individuals from the systemic inequalities embedded in institutions, policies, and social practices. As an umbrella cannot stop the rain [68], SES cannot fully mitigate the racial discrimination and structural barriers that Black people face. In fact, race is often more visible and salient than SES, meaning that even Black individuals at higher SES levels continue to experience significant discrimination and marginalization. Research shows that Black individuals with higher incomes and education levels often encounter more intense forms of discrimination, particularly in professional and social environments, as they challenge societal stereotypes. This persistent exposure to racism across SES levels undermines the potential protective effects of higher SES on health, brain development, and behavior, contributing to the Marginalization-Related Diminished Returns (MDRs) observed in the pathways from SES to outcomes such as brain structure and tobacco use. These realities underscore the need to address structural racism directly, as SES alone cannot counterbalance the entrenched racial inequalities that shape the lives of Black individuals [69-74].

### 4.1. Limitations

While this study offers important insights, several limitations should be noted. First, the ABCD study is ongoing, and the follow-up period, while substantial, is still limited to early adolescence. Longitudinal data extending into late adolescence and early adulthood will be critical for understanding the long-term effects of these pathways. Second, while we controlled for several key variables, unmeasured confounding factors such as community-level stressors or access to healthcare may also influence the observed associations. Finally, although the ABCD dataset is diverse, further research is needed to explore these pathways in other minority groups, such as Hispanic and Native American adolescents.

### 4.2. Future Directions

Future research should focus on policies and interventions aimed at undoing the effects of structural inequalities. This includes providing resources that can help equalize opportunities, such as improved educational systems, equitable access to healthcare, and support for families in low-SES communities. The role of peers, schools, and family environments should also be examined as potential buffers against the negative effects of low SES. Research should move beyond merely describing the problem of inequality to developing solution-based approaches that address the underlying social determinants of health and behavior.

### 4.3. Implications

The implications of this study are clear: addressing racial disparities in brain development and behavior requires more than improving SES alone. Policymakers and researchers must consider the broader social context, including the pervasive effects of structural racism, if they hope to reduce health disparities. Programs aimed at reducing tobacco use or improving adolescent brain health need to be tailored to account for the unique challenges faced by minority youth, particularly in terms of how SES-related benefits are constrained by systemic barriers.

## 5. Conclusion

In conclusion, this study highlights the importance of considering racial variation in the pathways from SES to brain development and behavior. Our findings support the MDRs framework, showing that Black adolescents experience weaker links between SES, brain structure, and tobacco use than their White peers. These results underscore the urgent need to address structural racism and to design interventions that can help equalize the effects of SES across diverse populations, ultimately promoting better health outcomes for all youth.

## Figures and Tables

**Table 1.** Descriptive Data Overall

	Mean	Std. Err. (SE)	[95% conf.	interval]
Age	9.480	0.005	9.470	9.490
	%	SE	[95% conf.	interval]
Race				
White	0.729	0.006	0.717	0.740
Black	0.271	0.006	0.260	0.283
Gender				
Female	0.476	0.007	0.463	0.489
Male	0.524	0.007	0.511	0.537
Marital Status of the Household				
Unwed Household	0.340	0.006	0.327	0.352
Married Household	0.660	0.006	0.648	0.673

**Table 2.** Summary of multigroup structural equation model

		B	SE	95%	CI	p
Racialized as White						
Outcome	Predictor					
Tobacco Use Initiation						
	Total Cortical Volume	−0.028	0.012	−0.053	−0.004	0.022
	Age	0.087	0.011	0.065	0.108	< 0.001
	Male	−0.008	0.012	−0.033	0.016	0.493
	Income to Need Ratio	−0.032	0.012	−0.054	−0.009	0.006
	Low Neighborhood Income	−0.015	0.011	−0.037	0.008	0.197
Total Cortical Volume						
	Age	−0.026	0.010	−0.045	−0.007	0.008
	Male	0.450	0.009	0.433	0.467	< 0.001
	Income to Need Ratio	0.091	0.010	0.071	0.110	< 0.001
	Married Household	0.089	0.010	0.070	0.108	< 0.001
	Low Neighborhood Income	−0.067	0.010	−0.087	−0.048	< 0.001
Racialized as Black						
Tobacco Use Initiation						
	Total Cortical Volume	−0.004	0.023	−0.048	0.040	0.865
	Age	0.038	0.020	−0.002	0.077	0.063
	Male	−0.002	0.022	−0.045	0.042	0.931
	Income to Need Ratio	0.023	0.022	−0.020	0.067	0.290
	Low Neighborhood Income	−0.015	0.021	−0.056	0.027	0.482
Total Cortical Volume						
	Age	−0.075	0.018	−0.111	−0.040	< 0.001
	Male	0.413	0.017	0.380	0.445	< 0.001
	Income to Need Ratio	0.129	0.020	0.089	0.168	< 0.001
	Married Household	0.090	0.018	0.054	0.126	< 0.001
	Low Neighborhood Income	−0.009	0.019	−0.048	0.029	0.626

## References

[1] Noble KG, Engelhardt LE, Brito NH, Socioeconomic disparities in neurocognitive development in the first two years of life. Dev Psychobiol. Jul 2015;57(5):535–51. doi:10.1002/dev.21303 PMC4821066 25828052

[2] Noble KG, Giebler MA. The Neuroscience of Socioeconomic Inequality. Curr Opin Behav Sci. Dec 2020;36:23–28. doi:10.1016/j.cobeha.2020.05.007 32719820 PMC7384696

[3] Noble KG, Houston SM, Kan E, Sowell ER. Neural correlates of socioeconomic status in the developing human brain. Dev Sci. Jul 2012;15(4):516–27. doi:10.1111/j.1467-7687.2012.01147.x 22709401 PMC6554027

[4] Noble KG, McCandliss BD. Reading development and impairment: behavioral, social, and neurobiological factors. J Dev Behav Pediatr. Oct 2005;26(5):370–8. doi:10.1097/00004703-200510000-00006 16222178

[5] Noble KG, McCandliss BD, Farah MJ. Socioeconomic gradients predict individual differences in neurocognitive abilities. Dev Sci. Jul 2007;10(4):464–80. doi:10.1111/j.1467-7687.2007.00600.x 17552936

[6] Ursache A, Noble KG. Neurocognitive development in socioeconomic context: Multiple mechanisms and implications for measuring socioeconomic status. Psychophysiology. Jan 2016;53(1):71–82. doi:10.1111/psyp.12547 26681619 PMC4685721

[7] Kwate NO. Fried chicken and fresh apples: racial segregation as a fundamental cause of fast food density in black neighborhoods. Health Place. Mar 2008;14(1):32–44. doi:10.1016/j.healthplace.2007.04.001 17576089

[8] Link BG, Phelan J. Social conditions as fundamental causes of disease. J Health Soc Behav. 1995;Spec No:80–94. 7560851

[9] Phelan JC, Link BG. Fundamental cause theory. Medical sociology on the move. Springer; 2013:105–125.

[10] Phelan JC, Link BG, Diez-Roux A, Kawachi I, Levin B. “Fundamental causes” of social inequalities in mortality: a test of the theory. J Health Soc Behav. Sep 2004;45(3):265–85. doi:10.1177/002214650404500303 15595507

[11] Mirowsky J, Ross CE. Education, cumulative advantage, and health. Ageing International. 2005;30(1):27–62.

[12] Mirowsky J, Ross CE. Education, Health, and the Default American Lifestyle. J Health Soc Behav. Sep 2015;56(3):297–306. doi:10.1177/0022146515594814 26272989

[13] Ross CE, Mirowsky J. Refining the association between education and health: the effects of quantity, credential, and selectivity. Demography. 1999;36(4):445–460. 10604074

[14] Ross CE, Wu C-l. The links between education and health. American sociological review. 1995:719–745.

[15] Donkin A, Roberts J, Tedstone A, Marmot M. Family socio-economic status and young children's outcomes. Journal of Children's Services. 2014;9(2):83–95.

[16] Marmot M. Economic and social determinants of disease. Bull World Health Organ. 2001;79(10):988–9. 11693982 PMC2566682

[17] Marmot M. Social determinants of health inequalities. Lancet. Mar 19-25 2005;365(9464):1099–104. doi:10.1016/S0140-6736(05)71146-6 15781105

[18] Marmot M. The health gap: the challenge of an unequal world. The Lancet. 2015;386(10011):2442–2444. 10.1016/S0140-6736(15)00150-6 26364261

[19] Marmot M, Wilkinson R. Social determinants of health. Oup Oxford; 2005.

[20] Stringhini S, Carmeli C, Jokela M, Socioeconomic status, non-communicable disease risk factors, and walking speed in older adults: multi-cohort population based study. BMJ. Mar 23 2018;360:k1046. doi:10.1136/bmj.k1046 29572376 PMC5865179

[21] Cohen-Zimerman S, Kachian ZR, Krueger F, Gordon B, Grafman J. Corrigendum to “Childhood socioeconomic status predicts cognitive outcomes across adulthood following traumatic brain injury” [Neuropsychologia 124 (2019) 1-8]. Neuropsychologia. Nov 2019;134:107142. doi:10.1016/j.neuropsychologia.2019.107142 30611733

[22] Farah MJ. The Neuroscience of Socioeconomic Status: Correlates, Causes, and Consequences. Neuron. Sep 27 2017;96(1):56–71. doi:10.1016/j.neuron.2017.08.034 28957676

[23] Farah MJ. Socioeconomic status and the brain: prospects for neuroscience-informed policy. Nat Rev Neurosci. Jul 2018;19(7):428–438. doi:10.1038/s41583-018-0023-2 29867123

[24] Gianaros P. Socioeconomic health disparities: A health neuroscience and lifecourse perspective. Psychological Science Agenda. 2011;25(1):345–356.

[25] Gianaros PJ, Hackman DA. Contributions of neuroscience to the study of socioeconomic health disparities. Psychosom Med. Sep 2013;75(7):610–5. doi:10.1097/PSY.0b013e3182a5f9c1 23975944 PMC3816088

[26] Gianaros PJ, Manuck SB. Neurobiological pathways linking socioeconomic position and health. Psychosom Med. Jun 2010;72(5):450–61. doi:10.1097/PSY.0b013e3181e1a23c 20498294 PMC2903752

[27] Hao Y, Farah MJ. The affective neuroscience of socioeconomic status: implications for mental health. BJPsych Bull. Jul 2 2020;44(5):1–6. doi:10.1192/bjb.2020.69 PMC7525591 32611462

[28] Madhushanthi HJ, Wimalasekera SW, Goonewardena CSE, Amarasekara A, Lenora J. Socioeconomic status is a predictor of neurocognitive performance of early female adolescents. Int J Adolesc Med Health. Jun 13 2018;32(6)doi:10.1515/ijamh-2018-0024 29897881

[29] Bissett PG, Hagen MP, Jones HM, Poldrack RA. Design issues and solutions for stop-signal data from the Adolescent Brain Cognitive Development (ABCD) study. Elife. 2021;10:e60185. 33661097 10.7554/eLife.60185 PMC7997655

[30] Casey BJ, Cannonier T, Conley MI, The Adolescent Brain Cognitive Development (ABCD) study: Imaging acquisition across 21 sites. Dev Cogn Neurosci. Aug 2018;32:43–54. doi:10.1016/j.dcn.2018.03.001 29567376 PMC5999559

[31] Hatton S. Preview of the Adolescent Brain Cognitive Development (ABCD) Study Release 3.0. Biological Psychiatry. 2020;87(9):S110–S111.

[32] Lisdahl KM, Sher KJ, Conway KP, Adolescent brain cognitive development (ABCD) study: Overview of substance use assessment methods. Developmental cognitive neuroscience. 2018;32:80–96. 29559216 10.1016/j.dcn.2018.02.007 PMC6375310

[33] Luciana M, Bjork JM, Nagel BJ, Adolescent neurocognitive development and impacts of substance use: Overview of the adolescent brain cognitive development (ABCD) baseline neurocognition battery. Developmental cognitive neuroscience. 2018;32:67–79. 29525452 10.1016/j.dcn.2018.02.006 PMC6039970

[34] Simmons C, Conley MI, Gee DG, Responsible Use of Open-Access Developmental Data: The Adolescent Brain Cognitive Development (ABCD) Study. Psychol Sci. Jun 2021;32(6):866–870. doi:10.1177/09567976211003564 34043478 PMC8726588

[35] Assari S. Parental Education, Household Income, and Cortical Surface Area among 9-10 Years Old Children: Minorities' Diminished Returns. Brain Sci. Dec 9 2020;10(12)doi:10.3390/brainsci10120956 PMC7763341 33317053

[36] Assari S, Akhlaghipour G, Saqib M, Boyce S, Bazargan M. Prefrontal Cortex Response to Threat: Race by Age Variation in 9-10 Year Old Children. Journal of Mental Health & Clinical Psychology. 2020;4(4):1–12. 33241232 10.29245/2578-2959/2020/4.1209

[37] Assari S, Boyce S. Race, Socioeconomic Status, and Cerebellum Cortex Fractional Anisotropy in Pre-Adolescents. Adolescents. Jun 2021;1(2):70–94. doi:10.3390/adolescents1020007 34095893

[38] Assari S, Boyce S, Bazargan M, Parental Educational Attainment, the Superior Temporal Cortical Surface Area, and Reading Ability among American Children: A Test of Marginalization-Related Diminished Returns. Children (Basel). May 18 2021;8(5)doi:10.3390/children8050412 PMC8158386 34070118

[39] Assari S, Sheikhattari P. Racialized influence of parental education on adolescents’ tobacco and marijuana initiation: Mediating effects of average cortical thickness. Journal of Medicine, Surgery, and Public Health. 2024/08/01/ 2024;3:100107. doi:10.1016/j.glmedi.2024.100107

[40] Darvishi M, Saqib M, Assari S. Diminished association between parental education and parahippocampal cortical thickness in pre-adolescents in the US. Stud Soc Sci Res. 2021;2:34–63.

[41] Assari S, Boyce S, Caldwell CH, Bazargan M. Parent Education and Future Transition to Cigarette Smoking: Latinos' Diminished Returns. Front Pediatr. 2020;8:457. doi:10.3389/fped.2020.00457 32974240 PMC7466764

[42] Assari S, Caldwell C, Bazargan M. Parental educational attainment and relatives' substance use of American youth: Hispanics Diminished Returns. J Biosci Med (Irvine). Feb 2020;8(2):122–134. doi:10.4236/jbm.2020.82010 32123689 PMC7051012

[43] Assari S, Cobb S, Saqib M, Bazargan M. Diminished Returns of Educational Attainment on Heart Disease among Black Americans. Open Cardiovasc Med J. 2020;14:5–12. doi:10.2174/1874192402014010005 32399080 PMC7217286

[44] Assari S, Najand B, Sheikhattari P. Household Income and Subsequent Youth Tobacco Initiation: Minorities’ Diminished Returns. Journal of Medicine, Surgery, and Public Health. 2024/02/02/ 2024:100063. doi:10.1016/j.glmedi.2024.100063 38425566 PMC10900246

[45] Assari S, Sheikhattari P, Zare H. Blacks’ Diminished Returns of Parental Education on Household Income: A Study of College Students in the US. Open Journal of Educational Research. 2024:187–196. 10.31586/ojer.2024.1016 PMC11981232 40206500

[46] Assari S, Zare H. Unequal Effect of Educational Attainment on Reducing Poverty and Welfare; Diminished Returns of American Indian/Alaska Native Populations. J Rehabil Ther. 2024;6(2):1–11. doi:10.29245/2767-5122/2024/2.1143 PMC11296615 39100915

[47] Assari S BM, Caldwell CH, Zimmerman MA. Diminished Returns of Parental Educational Attainment on School Achievement of Non-Hispanic Black High School Students. Under review. 2020; 10.3389/feduc.2020.00030 32596626

[48] Assari SA G, Boyce S, Bazargan M, Caldwell C,. Parental Human Capital and Adolescents’ Executive Function: Immigrants’ Diminished Returns. . Medical Research Archives. 2020;8(10):1–20. doi:10.18103/mra.v8i10.2235 PMC7695233 33251336

[49] Boyce S, Bazargan M, Caldwell CH, Zimmerman MA, Assari S. Parental Educational Attainment and Social Environment of Urban Public Schools in the U.S.: Blacks’ Diminished Returns. Children. 2020;7(5):44. 32397657 10.3390/children7050044 PMC7278682

[50] Boyce S, Darvishi M, Marandi R, Review Paper Racism-Related Diminished Returns of Socioeconomic Status on Adolescent Brain and Cognitive Development.

[51] S A. Minorities Diminished Returns. MedLine Publications. 2020;

[52] S A. Parental Educational Attainment and Frequency of Marijuana Use in Youth: Hispanics’ Diminished Returns. Journal of Education and Culture Studies 5(6):p47. 2021;5(6)doi:DOI: 10.22158/jecs.v5n6p47

[53] Assari S, Boyce S. Family's Subjective Economic Status and Children's Matrix Reasoning: Blacks' Diminished Returns. Res Health Sci. Nov 29 2021;6(1):1–23. doi:10.22158/rhs.v6n1p1 33299959

[54] Assari S, Boyce S, Akhlaghipour G, Bazargan M, Caldwell CH. Reward Responsiveness in the Adolescent Brain Cognitive Development (ABCD) Study: African Americans’ Diminished Returns of Parental Education. Brain Sciences. 2020;10(6):391. 32575523 10.3390/brainsci10060391 PMC7349244

[55] Assari S, Boyce S, Bazargan M, Caldwell CH. Diminished Returns of Parental Education in Terms of Youth School Performance: Ruling out Regression toward the Mean. Children. 2020;7(7):74. 32645933 10.3390/children7070074 PMC7401872

[56] Assari S, Boyce S, Bazargan M, Caldwell CH. African Americans’ diminished returns of parental education on adolescents’ depression and suicide in the Adolescent Brain Cognitive Development (ABCD) study. European journal of investigation in health, psychology and education. 2020;10(2):656–668. 32656052 10.3390/ejihpe10020048 PMC7351357

[57] Assari S, Boyce S, Bazargan M, Caldwell CH. Mathematical Performance of American Youth: Diminished Returns of Educational Attainment of Asian-American Parents. Educ Sci (Basel). Feb 2020;10(2) PMC7083587 32201681

[58] Assari S, Boyce S, Caldwell CH, Bazargan M. Minorities’ Diminished Returns of Parental Educational Attainment on Adolescents’ Social, Emotional, and Behavioral Problems. Children. 2020;7(5):49. 32443584 10.3390/children7050049 PMC7278850

[59] Galea S, Nandi A, Vlahov D. The social epidemiology of substance use. Epidemiologic reviews. 2004;26(1):36–52. 15234946 10.1093/epirev/mxh007

[60] David A, Esson K, Perucic A-M, Fitzpatrick C. Tobacco use: equity and social determinants. Equity, social determinants and public health programmes. 2010;199:218.

[61] Hudmon KS, Kilfoy BA, Prokhorov AV. The epidemiology of tobacco use and dependence. Critical Care Nursing Clinics. 2006;18(1):1–11. 16546003 10.1016/j.ccell.2005.10.002

[62] Gee GC, Ford CL. STRUCTURAL RACISM AND HEALTH INEQUITIES: Old Issues, New Directions. Du Bois Rev. Apr 2011;8(1):115–132. doi:10.1017/S1742058X11000130 25632292 PMC4306458

[63] Gee GC, Ford CL. Structural racism and health inequities: Old issues, New Directions1. Du Bois review: social science research on race. 2011;8(1):115–132. 25632292 10.1017/S1742058X11000130 PMC4306458

[64] Gee GC, Hicken MT. Structural racism: the rules and relations of inequity. Ethnicity & Disease. 2021;31(Suppl 1):293. 34045831 10.18865/ed.31.S1.293 PMC8143846

[65] Gee GC, Hing A, Mohammed S, Tabor DC, Williams DR. Racism and the Life Course: Taking Time Seriously. Am J Public Health. Jan 2019;109(S1):S43–S47. doi:10.2105/AJPH.2018.304766 30699016 PMC6356137

[66] Gee GC, Walsemann KM, Brondolo E. A life course perspective on how racism may be related to health inequities. Am J Public Health. May 2012;102(5):967–74. doi:10.2105/AJPH.2012.300666 22420802 PMC3483932

[67] Paradies Y, Ben J, Denson N, Racism as a Determinant of Health: A Systematic Review and Meta-Analysis. PLoS One. 2015;10(9):e0138511. doi:10.1371/journal.pone.0138511 26398658 PMC4580597

[68] Hamilton D, Darity W Jr, Price AE, Sridharan V, Tippett R. Umbrellas don’t make it rain: Why studying and working hard isn’t enough for Black Americans. New York: The New School. 2015;

[69] Hudson D, Sacks T, Irani K, Asher A. The price of the ticket: health costs of upward mobility among African Americans. International journal of environmental research and public health. 2020;17(4):1179. 32069785 10.3390/ijerph17041179 PMC7068450

[70] Hudson DL, Bullard KM, Neighbors HW, Geronimus AT, Yang J, Jackson JS. Are benefits conferred with greater socioeconomic position undermined by racial discrimination among African American men? Journal of men's health. 2012;9(2):127–136. 10.1016/j.jomh.2012.03.006 PMC3371660 22707995

[71] Hudson DL, Eaton J, Lewis P, Grant P, Sewell W, Gilbert K. “Racism?!?... Just look at our neighborhoods” Views on racial discrimination and coping among African American men in Saint Louis. The Journal of Men’s Studies. 2016;24(2):130–150. 10.1177/1060826516641103 PMC7581294 33100801

[72] Hudson DL, Neighbors HW, Geronimus AT, Jackson JS. The relationship between socioeconomic position and depression among a US nationally representative sample of African Americans. Soc Psychiatry Psychiatr Epidemiol. Mar 2012;47(3):373–81. doi:10.1007/s00127-011-0348-x 21293845 PMC3279642

[73] Hudson DL, Neighbors HW, Geronimus AT, Jackson JS. Racial Discrimination, John Henryism, and Depression Among African Americans. J Black Psychol. Jun 2016;42(3):221–243. doi:10.1177/0095798414567757 27529626 PMC4903152

[74] Hudson DL, Puterman E, Bibbins-Domingo K, Matthews KA, Adler NE. Race, life course socioeconomic position, racial discrimination, depressive symptoms and self-rated health. Soc Sci Med. Nov 2013;97:7–14. doi:10.1016/j.socscimed.2013.07.031 24161083

